# Hydrogen Sulfide, an Emerging Regulator of Acid-Sensing Ion Channels

**DOI:** 10.1093/function/zqab014

**Published:** 2021-03-15

**Authors:** Csaba Szabo

**Affiliations:** Chair of Pharmacology, Faculty of Science and Medicine, University of Fribourg, Fribourg, Switzerland

## A Perspective on “Hydrogen Sulfide Upregulates Acid-Sensing Ion Channels via the MAPK-Erk1/2 Signaling Pathway”

In response to intensive research efforts conducted over the last two decades, hydrogen sulfide (H_2_S) has been “promoted” from a toxic gas and environmental hazard to an important endogenous mammalian biological regulator.^1^ Currently, we consider three enzymes, cystathionine-β-synthase (CBS), cystathionine γ-lyase (CSE), and 3-mercaptopyruvate sulfurtransferase (3-MST) as the main sources of H_2_S in most mammalian cells and tissues, with several additional enzymes—as well as nonenzymatic reactions and H_2_S-producing bacteria in the bacterial microbiota—also contributing.^2^^,^^3^ H_2_S, once produced, can travel significant distances within and between different cell types, and can act as an autocrine and paracrine messenger. Its physiological roles include the regulation of cardiovascular, neuronal, metabolic, and immune function; its pathophysiological roles range from cardiovascular disease to inflammation, ischemia, neurodegeneration, cancer, and many others.^2^^,^^3^

Membrane channels represent the important biological targets of H_2_S in mammalian cells. Starting with the discovery of Rui Wang demonstrating^4^ that K_ATP_ channels are biological targets for H_2_S and continuing with the delineation of H_2_S mode of action on this channel (which involves the sulfhydration of its Kir 6.1 subunit, followed by channel opening)^5^ a significant body of work focused on the effect of H_2_S on various membrane channels. Currently, at least a dozen channels are known to be regulated by H_2_S in direct or indirect manner, thereby regulating the transport of various ions including sodium, potassium, and calcium.^2^^,^^3^ Another class of H_2_S-related cellular regulatory effects relates to the action of this mediator on the activity of various signal transduction pathways, including the NF-κB pathway, the Akt pathway, various MAP kinases, Nrf-2, and many others; via these actions (as well as via a variety of additional intracellular mechanisms, including epigenetic regulatory effects), H_2_S acts as a global regulator of signal transduction and protein expression.^2^^,^^3^

The current report by Peng and Kellenberger published in “Function” identifies a novel group of channels, the acid-sensing ion channels (ASICs) as targets for H_2_S-mediated modulation.^6^ ASICs are low pH-activated sodium-permeable ion channels, which are expressed on many cell types, both in the central and in the peripheral nervous system. As pH sensors, ASICs induce neuronal excitation under acidic conditions. ASICs have been implicated in the regulation of various physiological processes (eg, pain sensation, learning, and fear sensing); they have been also proposed to play a role in various pathophysiological processes (eg, stroke and neurodegeneration).^7^^,^^8^ Peng and Kellenberger report two types of regulatory mechanisms related to ASICs and H_2_S: an acute/direct one, related to a direct action of H_2_S on the ASICs, which leads to the potentiation of channel activity, and a longer-term one, whereby the expression of ASICs is increased, via an intracellular signaling process that likely depends on the effect of H_2_S on mitogen-activated protein kinases-Erk1/2 pathway. The effect of H_2_S on ASICs appears to be a “class effect” and it regulates a variety of ASICs including ASIC1a, ASIC1b, ASIC2a, and ASIC3.^6^ Another recent study, performed by an independent group of investigators, has also recently demonstrated the activating effect of H_2_S on ASICs.^9^ Based on various lines of studies presented in the report by Peng and Kellenberger,^6^ it was concluded that the longer-term, gene-transcription-dependent effect of H_2_S is probably more relevant in a physiological or pathophysiological context, than acute, direct effect of H_2_S on these channels.

Thus, ASICs are emerging as new class of biological targets for H_2_S. These channels—in addition to probably hundreds of previously identified molecular targets of H_2_S—may be involved in the actions of H_2_S in physiological conditions and in disease conditions in the central nervous system. Nevertheless, further studies remain to be conducted to determine if the H_2_S-mediated regulation of ASICs, indeed, occurs in the physiological context. Among many questions, the first one could be the following: Is the activity and/or expression of ASICs modulated by H_2_S, when this mediator is produced by endogenous enzymatic sources, such as CBS, CSE, and/or 3-MST. The body of evidence provided by Peng and Kellenberger study that relies on pharmacological (ie, exogenous) H_2_S donors, which are not ideal to mimic physiological H_2_S fluxes. (In this respect, the slow-acting H_2_S donor GYY4137, which was also employed by this study, and appears to show a bell-shaped concentration-response—is certainly a better tool than the more commonly used NaHS, which tends to create extremely high “peak” concentrations, followed by a rapid dissipation.^3^^,^^10^) Experiments using inhibitors or silencing of endogenous H_2_S-generating enzymes should be conducted in order to determine whether endogenously produced H_2_S exerts a regulatory role on the expression or activity of ASICs. Such pharmacological tools (with some limitations regarding their selectivity and/or potency) are available,^3^^,^^10^ and so are genetically modified mice lacking any one of the three major endogenous H_2_S-producing enzymes.^2^ Using such approaches, the physiological or pathophysiological role of H_2_S in the regulation of ASICs can be delineated in the future.

## Funding

The work of the author is supported by the Swiss National Science Foundation (SNSF).

## Author’s Contributions

C.S. conceived and wrote the manuscript.

## Conflict of Interest Statement

The author has no conflicts of interest to disclose.
